# GL-V9, a new synthetic flavonoid derivative, ameliorates DSS-induced colitis against oxidative stress by up-regulating Trx-1 expression via activation of AMPK/FOXO3a pathway

**DOI:** 10.18632/oncotarget.4657

**Published:** 2015-07-16

**Authors:** Yue Zhao, Yang Sun, Youxiang Ding, Xiaoping Wang, Yuxin Zhou, Wenjun Li, Shaoliang Huang, Zhiyu Li, Lingyi Kong, Qinglong Guo, Na Lu

**Affiliations:** ^1^ State Key Laboratory of Natural Medicines, Jiangsu Key Laboratory of Carcinogenesis and Intervention, Jiangsu Key Laboratory of Drug Design and Optimization, China Pharmaceutical University, Nanjing 210009, China; ^2^ School of Pharmacy, China Pharmaceutical University, Nanjing 210009, China; ^3^ State Key Laboratory of Natural Medicines, Department of Natural Medicinal Chemistry, China Pharmaceutical University, Nanjing 210009, China

**Keywords:** GL-V9, ROS, Trx-1, AMPK, colitis

## Abstract

GL-V9, a new synthesized flavonoid derivative, has been reported to possess anti-cancer properties in our previous studies. Uncontrolled overproduction of reactive oxygen species (ROS) has been implicated in oxidative damage of inflammatory bowel disease (IBD). In this study, we aimed to investigate the protective effect of GL-V9 against dextran sulfate sodium (DSS)-induced colitis. GL-V9 attenuated DSS-induced body weight loss, colon length shortening and colonic pathological damage. GL-V9 also inhibited inflammatory cells infiltration and decreased myeloperoxidase (MPO) and inducible nitric oxide synthase (iNOS) activities. Moreover, GL-V9 inhibited ROS and malondialdehyde (MDA) generation, but enhanced superoxide dismutase (SOD), glutathione (GSH) and total antioxidant capacity. GL-V9 reduced pro-inflammatory cytokines production in serum and colon as well. Mechanically, GL-V9 could increase Trx-1 via activation of AMPK/FOXO3a to suppress DSS-induced colonic oxidative stress. Furthermore, GL-V9 decreased pro-inflammatory cytokines and ROS production and increased the antioxidant defenses in the mouse macrophage cells RAW264.7 by promoting Trx-1 expression. In conclusion, our study demonstrated that GL-V9 attenuated DSS-induced colitis against oxidative stress by up-regulating Trx-1 via activation of AMPK/FOXO3a pathway, suggesting that GL-V9 might be a potential effective drug for colitis.

## INTRODUCTION

Ulcerative colitis (UC) is a chronic idiopathic inflammatory bowel disease (IBD) with multifactorial etiology [1, 2]. Although the pathophysiology of ulcerative colitis remains much debatable, increasing experimental and clinical evidence suggests that chronic gut inflammation may result from immune system dysfunction. This uncontrolled immune system activation results in the sustained overproduction of reactive oxygen species (ROS) with subsequent cellular oxidative stress damage [3–6], suggesting that ROS play a crucial role in the pathophysiology of IBD. Therefore, scavenging ROS is considered to be critical for regulating intestinal inflammation.

AMP-activated protein kinase (AMPK), a multisubstrate protein kinase consisting of three heterogenic subunits including a catalytic α-subunit and two regulatory β- and γ-subunits, is a key regulator in maintaining intracellular homoeostasis [7]. In recent years, intensive investigations indicate that AMPK not only functions as an intracellular energy sensor and regulator [8, 9], but is also a general stress sensor during many kinds of stress challenges [10–14]. It has been increasingly recognized that activation of AMPK pathway reduces intracellular ROS levels [15], suggesting that AMPK plays an important role in the regulation of cellular antioxidant defense. Another critical mediator of ROS homeostasis is the forkhead box subfamily O (FOXO). FOXO transcription factors are good candidates regulated by AMPK. The transcription factors FOXO1, FOXO3a, and FOXO4 have been implicated in many of the above ROS-regulated processes [16]. FOXO proteins promote oxidative stress resistance by binding to the promoters of the genes encoding SOD2, catalase and peroxiredoxin 3 [17–19].

Thioredoxin-1 (Trx-1), a 12 kDa dithiol protein possessing oxidoreductase activity, conserved from archaea and bacteria to man, is a key factor that maintains the protein dithiol/disulfide homeostasis. As a major antioxidant protein, Trx-1 is ubiquitously expressed and potently protects cells from oxidative damage by enhancing the catalytic activity of peroxiredoxin and glutathione peroxidase [20, 21], which plays a vital role in maintaining the cellular redox balance [22]. Trx-1 has been reported to protect cells from ROS-induced cytotoxicity [23]. Exogenous administration of recombinant human Trx-1 (rhTrx-1) can suppress 1-methyl-4-phenylpyridinium-induced neurotoxicity in the rat [24], brain damage caused by transient focal cerebral ischemia in mice, pro-inflammatory cytokine- or bleomycin-induced lung injury [25], and ethanol-or indomethacin-induced gastric mucosal injury [26]. Moreover, systemically overexpressed Trx-1 could prevent focal cerebral ischemia [27], retinal photo-oxidative damage [28], and renal ischemia/reperfusion injury [29]. It has been documented that rhTrx-1 has potent protective effect in experimental colitis as well [30]. Accordingly, Trx-1 is essential for regulating of redox signaling to protect cellular oxidative damage.

**Figure 1:**
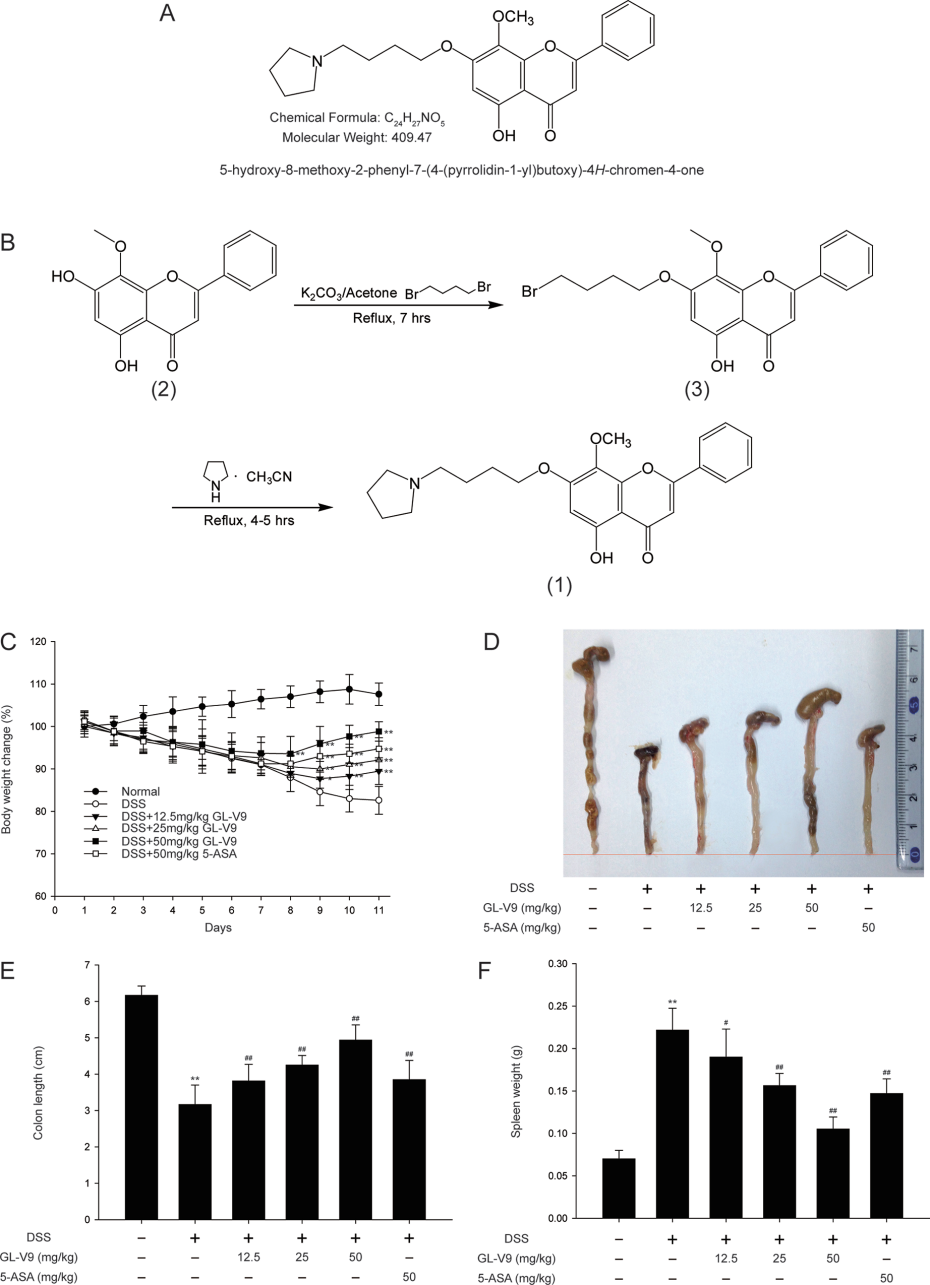
GL-V9 attenuated DSS-induced experimental colitis **A.** Chemical structure of GL-V9. **B.** Synthesis scheme of GL-V9. **C.** Body weight changes of each group (n = 8 per group) after DSS induction of colitis. **D.** Macroscopic appearances and **E.** the length of colons from each group of mice were measured. **F.** Spleen weight changes during the colitis process. Data are presented as mean ± SD. *p < 0.05, **p < 0.01 compared with normal mice; ^#^*p* < 0.05, ^##^*p* < 0.01 compared with DSS-treated colitis mice.

GL-V9 (5-hydroxy-8-methoxy-2-phenyl-7-(4-(pyrrolidin-1-yl)butoxy)4H-chromen-4-one) (Figure 1A) is a new synthesized flavonoid (Figure 1B), which has pro-apoptotic, anti-invasive and anti-metastatic effects [31, 32]. Wogonin exerts antioxidative, anti-inflammatory and anti-cancer activities [33–35], from which GL-V9 was synthesized. In this study, we study the effect of GL-V9 on ulcerative colitis, further to explore the potential therapeutic function of GL-V9 on inflammation.

## RESULTS

### GL-V9 attenuated DSS-induced colon injury and inflammatory symptoms

**Figure 2:**
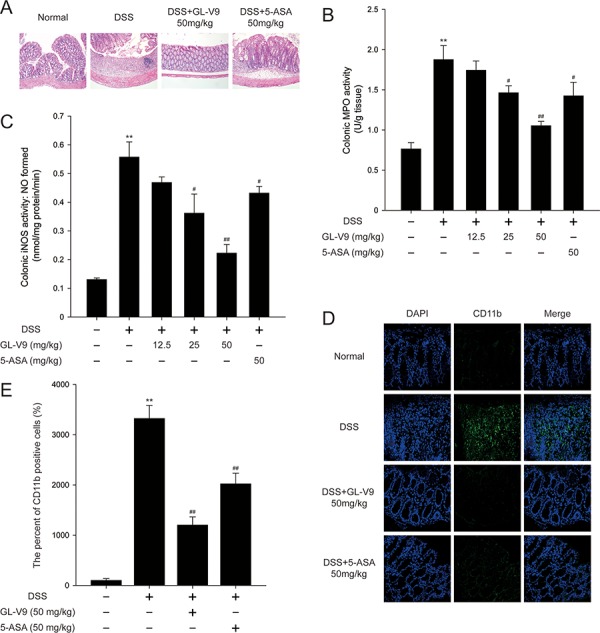
GL-V9 prevented DSS-induced colon damage in mice **A.** Serial sections of colon tissues were stained with hematoxylin and eosin (H&E). **B.** MPO and **C.** iNOS activities in the colonic tissues were detected. **D.** Sections of colon tissues were immunostained with DAPI (blue) and anti CD11b-FITC (green) and observed by confocal laser-scanning microscope. **E.** The CD11b expression was quantified with statistical significances. Data are presented as mean ± SD. **p < 0.01 compared with normal mice; ^#^*p* < 0.05, ^##^*p* < 0.01 compared with DSS-treated colitis mice.

In this study, we used the DSS-induced colitis in mice, a well-established preclinical model that exhibits many phenotypic features of relevance to human ulcerative colitis [36]. In general, DSS-induced colitis is characterized by a significant body weight loss. Our results showed that DSS-treated colitis mice exhibited dramatic body weight loss, while 50 mg/kg GL-V9 could remarkably ameliorate the loss of body weight after removing DSS, days 8–11 (Figure 1C). The colon was markedly shorter in DSS-treated mice than in GL-V9-treated mice (Figure 1D and 1E). Moreover, GL-V9 reversed DSS-induced gain of spleen weight (Figure 1F). We next evaluated the protective effect of GL-V9 by histopathological analysis using Haematoxylin & Eosin (H&E) staining (Figure 2A). Compared to the normal group, the DSS-induced colitis group exhibited marked erosion of the lamina propria mucosa, disappearance of glandular epithelium and inflammatory cell infiltration. Strikingly, 50 mg/kg GL-V9 could inhibit inflammatory cell infiltration and preserve intact colonic architecture with no obvious ulcer. Moreover, GL-V9 at 50 mg/kg notably suppressed DSS-induced myeloperoxidase (MPO) and inducible nitric oxide synthase (iNOS) activities (Figure 2B and 2C). It has been reported that CD11b is expressed on the surface of many leukocytes including monocytes, neutrophils, natural killer cells, granulocytes and macrophages [37]. Thus, we used CD11b^+^ as an indicator to monitor inflammation process and further determine the beneficial effect of GL-V9. We observed a great number of CD11b^+^ inflammatory cells accumulated at the mucosa of the lesion site in colonic tissues from DSS-treated mice. However, 50 mg/kg GL-V9 dramatically reduced the number of infiltrating CD11b^+^ inflammatory cells in colon tissues (Figure 2D and 2E). These results suggested that GL-V9 could successfully ameliorate DSS-induced colitis.

### GL-V9 diminished pro-inflammatory mediators production in the serum and colon of DSS-induced colitis mice

**Figure 3:**
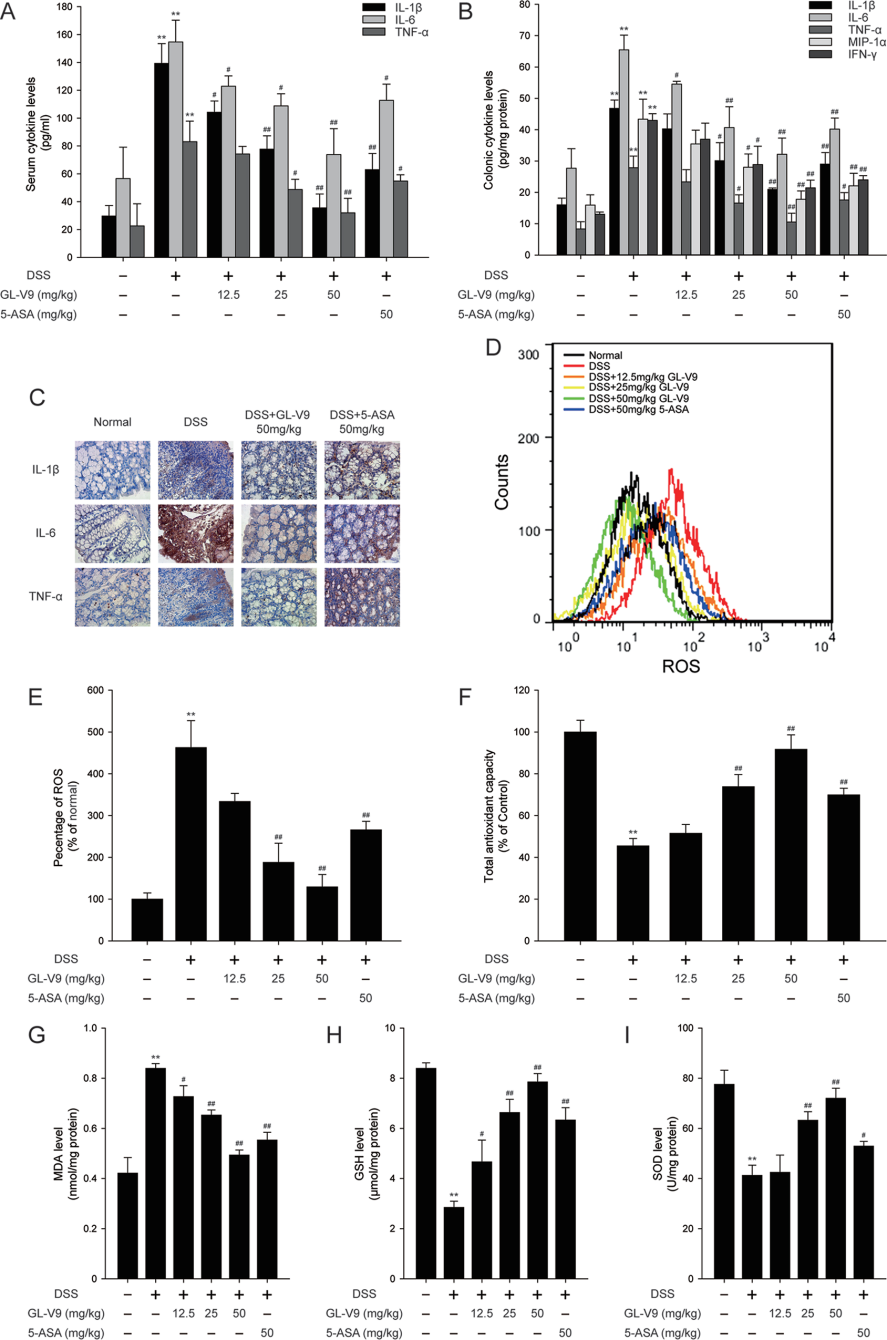
GL-V9 reduced pro-inflammatory cytokines production and enhanced the antioxidant defenses **A.** The production of inflammation-related cytokines IL-1β, IL-6 and TNF-α in serum and **B.** protein levels of cytokines including IL-1β, IL-6, TNF-α, MIP-1α and IFN-γ in colonic homogenate were determined by ELISA in triplicate. **C.** The expressions of IL-1β, IL-6 and TNF-α were detected by immunohistochemistry (×40) in colonic tissues. **D.** The colon tissue cells were loaded with DCFH/DA. The geometric mean DCF fluorescence was measured by flow cytometry. **E.** Histograms represented the geometric mean DCF fluorescence. Levels of the **F.** total antioxidant capacity, **G.** MDA, **H.** GSH and **I.** SOD were measured in colonic tissues. Data are presented as mean ± SD. **p < 0.01 compared with normal mice; ^#^*p* < 0.05, ^##^*p* < 0.01 compared with DSS-treated colitis mice.

Pro-inflammatory cytokines play a crucial role in the development of DSS-induced colitis [38]. As a chemotactic agent, MIP-1α contributes to the pathogenesis of IBD as well [39]. To gain an insight into the effect of GL-V9 on the inflammatory status of DSS-induced colitis, we assessed the levels of these inflammatory mediators. The production of IL-1β, IL-6 and TNF-α in the serum were significantly increased after DSS challenge. However, GL-V9 remarkably inhibited the elevated levels of these cytokines (Figure 3A). We also measured the levels of IL-1β, IL-6, TNF-α, MIP-1α and IFN-γ in colonic homogenates. GL-V9 notably suppressed DSS-induced high production of IL-1β, IL-6, TNF-α, MIP-1α and IFN-γ (Figure 3B). Furthermore, GL-V9 at 50 mg/kg significantly inhibited the increased number of IL-1β-, IL-6- and TNF-α-positive cells in colonic mucosa of DSS-induced mice (Figure 3C).

### GL-V9 counteracted DSS-induced oxidative stress by enhancing the antioxidant defenses

During active episodes of IBD, the uncontrolled overproduction of ROS could cause oxidative damage to cells and tissues, suggesting that the process of colonic inflammation and oxidative stress are tightly linked [40]. Thus, we investigated whether GL-V9 ameliorated DSS-induced colitis via inhibiting oxidative stress. Strikingly, GL-V9 decreased ROS generation in colon tissues of DSS-induced colitis mice (Figure 3D and 3E). We determined DSS-induced oxidative stress by measuring the levels of lipid peroxides expressed as MDA in colon tissues of colitis mice. We found that DSS induced elevated malondialdehyde (MDA), while GL-V9 effectively counteracted the above changes. Moreover, we assessed the colonic levels of antioxidant defenses such as superoxide dismutases (SOD), Glutathione (GSH) and total antioxidant capacity (TAC) (Figure 3F–3I). Colonic SOD, GSH and total antioxidant capacity were dramatically reduced in DSS-treated mice, but these reductions were restored by GL-V9 treatment to levels near to the normal control values. Our findings indicated the potential efficacy of GL-V9 in mitigating oxidative stress and boosting the antioxidant defenses in DSS-induced colitis.

### GL-V9 increased antioxidant Trx-1 via activation of AMPK/FOXO3a pathway in DSS-colitis mice

**Figure 4:**
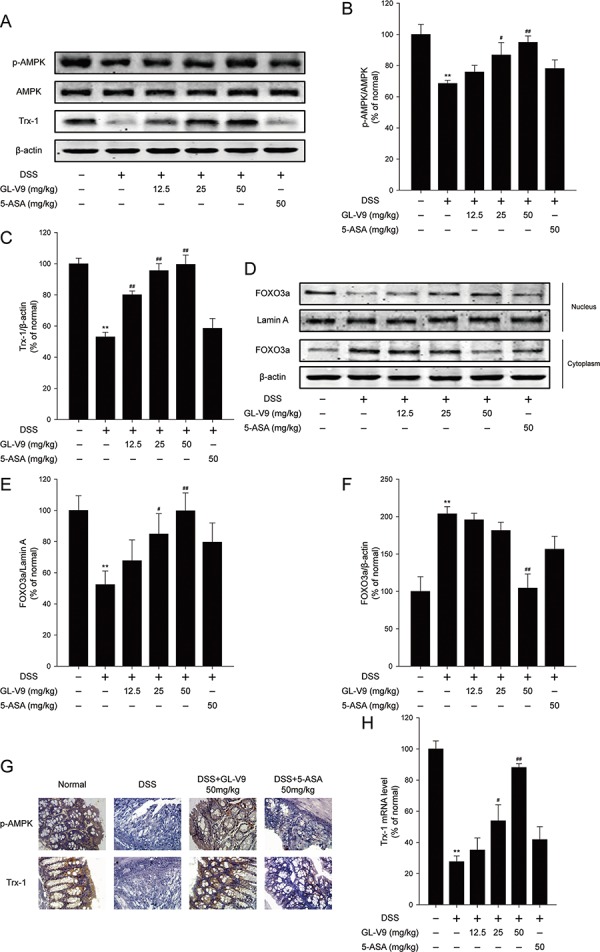
GL-V9 increased Trx-1 expression by activating AMPK/FOXO3a signaling pathway in DSS-colitis mice **A.** The levels of p-AMPK, AMPK and Trx-1 were assessed by Western Blot in colonic tissues. **B.** and **C.** Densitometric analysis was performed to determine the relative ratios of each protein. **D.** FOXO3a nuclear translocation was determined by Western Blot in colonic tissues. **E.** and **F.** Densitometric analysis was performed to determine the relative ratios of each protein. Lamin A and β-actin were used as nuclear and cytoplasmic markers, respectively. **G.** The expressions of p-AMPK and Trx-1 were detected by immunohistochemistry (×40) in colonic tissues. **H.** Trx-1 mRNA level was measured by real-time PCR in colonic tissues. Data are presented as mean ± SD. **p < 0.01 compared with normal mice; ^#^*p* < 0.05, ^##^*p* < 0.01 compared with DSS-treated colitis mice.

Activation of AMP-activated protein kinase (AMPK) pathway and the thioredoxin (Trx) system could reduce intracellular reactive oxygen species (ROS) levels [15]. The previous study has shown that AMPK increased Trx transcription by increasing the nuclear translocation of FOXO3 [41]. We therefore determined whether GL-V9 exerted the antioxidative effect by up-regulating Trx-1 via activation of AMPK/FOXO3a pathway. As expected, GL-V9 remarkably up-regulated phosphorylated-AMPK and Trx-1 expression and increased nuclear translocation of FOXO3a in the colon tissues of DSS-induced colitis mice (Figure 4A–4F). The findings were further confirmed by IHC staining of phosphorylated-AMPK and Trx-1. We found that 50 mg/kg GL-V9 strikingly increased levels of phosphorylated-AMPK and Trx-1 in the colon tissues of DSS-induced colitis mice (Figure 4G). Moreover, GL-V9 increased the mRNA level of Trx-1 in the colonic tissues of DSS-treated mice (Figure 4H). These results suggested that GL-V9 could increase Trx-1 expression via activation of AMPK/FOXO3a pathway.

### GL-V9 inhibited pro-inflammatory cytokines and enhanced the antioxidant defenses *in vitro*

**Figure 5:**
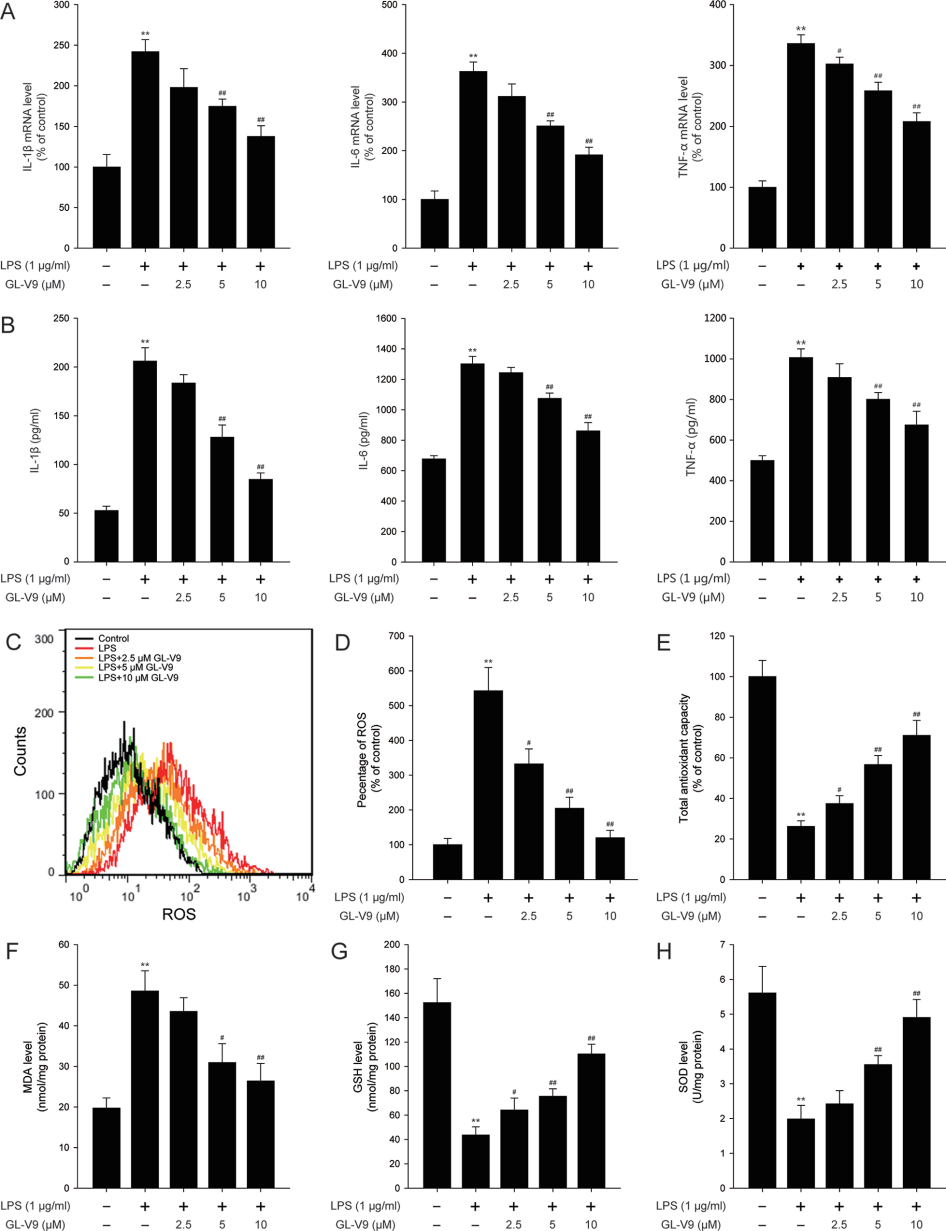
GL-V9 inhibited pro-inflammatory cytokines production and enhanced the antioxidant defenses in LPS-induced RAW 264.7 cells **A.** The mRNA levels of IL-1β, IL-6 and TNF-α were measured by real-time PCR following treatment with 1 μg/ml LPS and 2.5, 5, 10 μM GL-V9 for 12 h in RAW 264.7 cells. **B.** IL-1β, IL-6 and TNF-α secreted into culture supernatants were measured by ELISA following treatment with 1 μg/ml LPS and 2.5, 5, 10 μM GL-V9 for 12 h in RAW 264.7 cells. **C.** The RAW 264.7 cells were loaded with DCFH/DA. The geometric mean DCF fluorescence was measured by flow cytometry. **D.** Histograms represented the geometric mean DCF fluorescence. Levels of the **E.** total antioxidant capacity, **F.** MDA, **G.** GSH and **H.** SOD were measured following treatment with 1 μg/ml LPS and 2.5, 5, 10 μM GL-V9 for 12 h in RAW 264.7 cells. Data are presented as mean ± SD. **p < 0.01 compared with control group; ^#^*p* < 0.05, ^##^*p* < 0.01 compared with LPS-treated group.

The pro-inflammatory cytokines such as IL-1β, IL-6 and TNF-α play key roles in inflammation-related diseases. Our *in vivo* study demonstrated that GL-V9 inhibited the secretion of IL-1β, IL-6 and TNF-α (Figure 3). To confirm our conclusion *in vivo*, we investigated the effect of GL-V9 in the mouse macrophage cell line RAW264.7. As expected, GL-V9 inhibited LPS-induced increased mRNA levels and high production of IL-1β, IL-6 and TNF-α (Figure 5A and 5B). Moreover, GL-V9 significantly suppressed ROS production induced by LPS (Figure 5C and 5D). Then, we found that levels of SOD, GSH and total antioxidant capacity were decreased and MDA levels were increased in LPS-treated RAW 264.7 cells, but these effects were reversed by GL-V9 treatment (Figure 5E and 5H). These results demonstrated that GL-V9 inhibited pro-inflammatory cytokines and enhanced the antioxidant defenses *in vitro*.

### GL-V9 increased antioxidant Trx-1 via activation of AMPK/FOXO3a pathway *in vitro*

**Figure 6:**
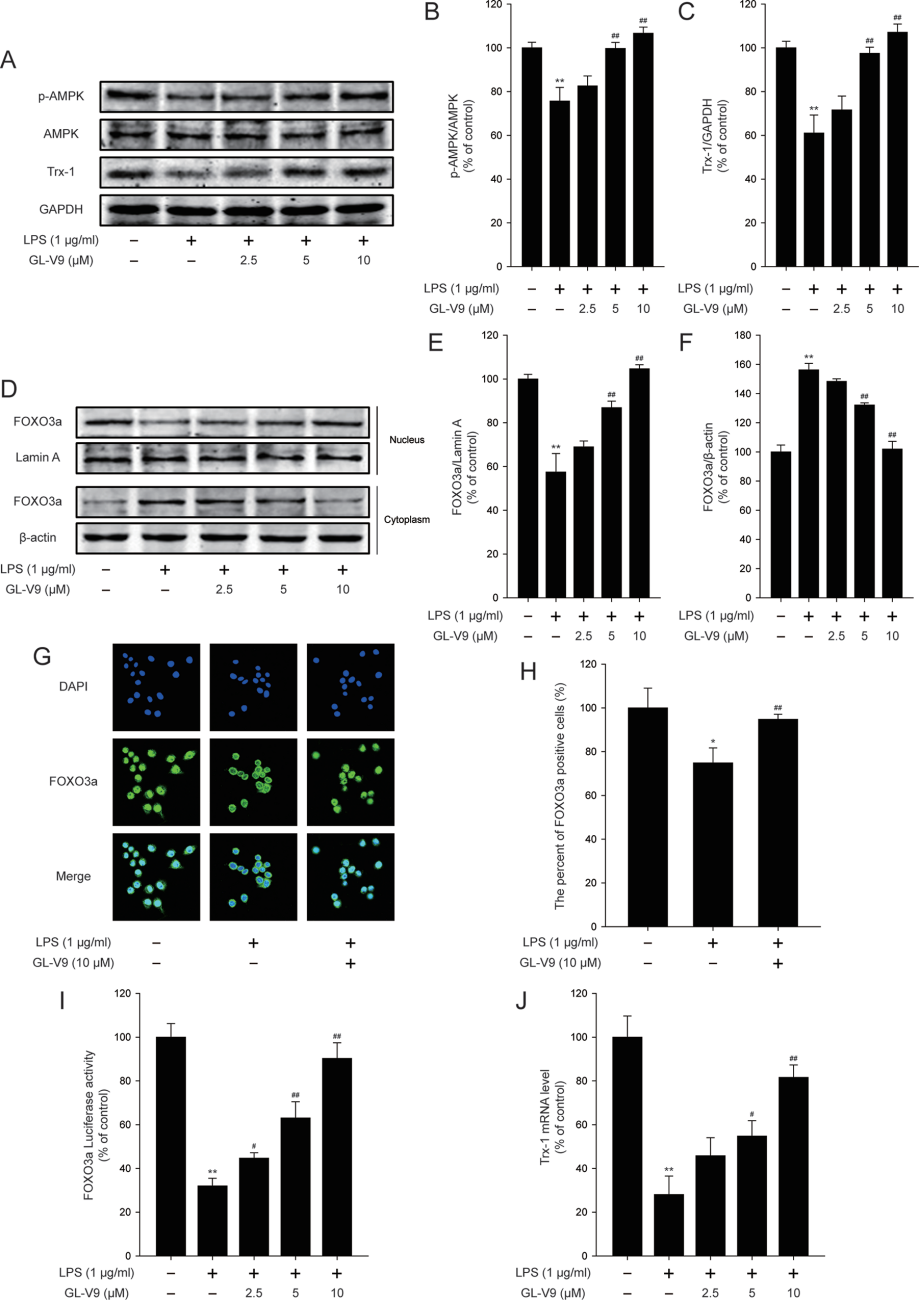
GL-V9 increased Trx-1 expression by activating AMPK/FOXO3a signaling pathway in LPS-induced RAW 264.7 cells RAW 264.7 cells were treated with 1 μg/ml LPS alone or with indicated concentrations of GL-V9 for 12 h. **A.** Levels of p-AMPK, AMPK and Trx-1 were assessed by Western Blot. **B.** and **C.** Densitometric analysis was performed to determine the relative ratios of each protein. **D.** FOXO3a nuclear translocation was determined by Western Blot. **E.** and **F.** Densitometric analysis was performed to determine the relative ratios of each protein. Lamin A and β-actin were used as nuclear and cytoplasmic markers, respectively. **G.** Immunofluorescence staining of FOXO3a was detected to determine the effect of GL-V9 on FOXO3a nuclear translocation (image magnification: 400×). **H.** The FOXO3a expression was quantified with statistical significances. **I.** To examine the transcriptional activities of FOXO3a, RAW 264.7 cells were cotransfected with FOXO-luc and pRL-TK Renilla. Luciferase activity was determined 12 h posttreatment by promega dual luciferase reporter assay system, normalized against values for the corresponding pRL-TK Renilla activity. **J.** Trx-1 mRNA level was measured by real-time PCR. Data are presented as mean ± SD. *p < 0.05, **p < 0.01 compared with control group; ^#^*p* < 0.05, ^##^*p* < 0.01 compared with LPS-treated group.

**Figure 7:**
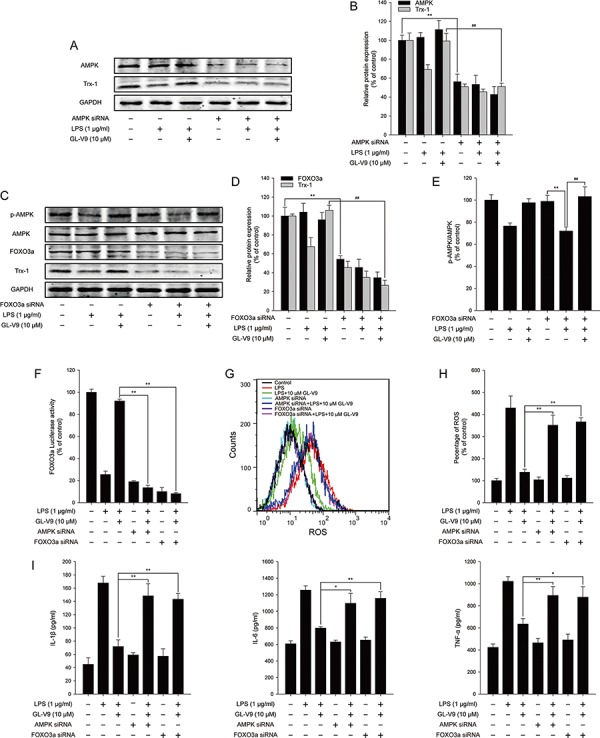
AMPK/FOXO3a/Trx-1 signaling pathway was involved in the anti-inflammatory effect of GL-V9 in LPS-induced RAW 264.7 cells AMPK siRNA and FOXO3a siRNA were transfected into RAW 264.7 cells respectively. The cells were cultured in serum free medium. After 6 h, cells were treated with 1 μg/ml LPS and 10 μM GL-V9 for 12 h. **A.** AMPK and Trx-1 were detected by Western Blot after transfection of AMPK siRNA. **B.** The relative ratios of AMPK and Trx-1 protein were represented by densitometric analysis. **C.** p-AMPK, AMPK, FOXO3a and Trx-1 were detected by Western Blot after transfection of FOXO3a siRNA. **D.** and **E.** The relative ratios of p-AMPK, FOXO3a and Trx-1 protein were represented by densitometric analysis. **F.** The transcriptional activities of FOXO3a in RAW 264.7 cells cotransfected with FOXO-luc and pRL-TK Renilla with LPS and GL-V9. **G.** After LPS and GL-V9 treatment, the RAW 264.7 cells were loaded with DCFH/DA. The geometric mean DCF fluorescence was measured by flow cytometry. **H.** Histograms represented the geometric mean DCF fluorescence. **I.** IL-1β, IL-6 and TNF-α secreted into culture supernatants were measured by ELISA. Data are presented as mean ± SD. *p < 0.05 **p < 0.01.

To elucidate the antioxidative mechanism of GL-V9, we examined the effect of GL-V9 on the activation of AMPK/FOXO3a/Trx-1 pathway *in vitro*. Interestingly, GL-V9 increased phosphorylated-AMPK and Trx-1 expression and increased nuclear translocation of FOXO3a in RAW 264.7 cells stimulated with LPS (Figure 6A–6H). The Luciferase reporter gene assay revealed that GL-V9 increased transcriptional activity of FOXO3a (Figure 6I). GL-V9 could remarkably increase the transcriptional expression of Trx-1 as well (Figure 6J). To further verify the role of AMPK/FOXO3a in antioxidative mechanism of GL-V9, we transfected RAW 264.7 cells with AMPK siRNA or FOXO3a siRNA. After transfection with AMPK siRNA, the endogenous AMPK was knocked down (Figure 7A and 7B). Trx-1 protein expression increased by GL-V9 was withdrawn by AMPK siRNA transfection (Figure 7A and 7B). FOXO3a siRNA reduced FOXO3a protein efficiently (Figure 7C and 7D). FOXO3a siRNA transfection inhibited GL-V9-induced Trx-1 expression as well (Figure 7C and 7D). However, the effect of GL-V9 on AMPK activation could not be blocked by FOXO3a siRNA transfection (Figure 7C and 7E). AMPK siRNA and FOXO3a siRNA transfection reversed increased transcriptional activity of FOXO3a by GL-V9, respectively (Figure 7F). Furthermore, the inhibitory effects of GL-V9 on LPS-induced ROS generation and pro-inflammatory cytokines production such as IL-1β, IL-6 and TNF-α were remarkably attenuated by AMPK siRNA or FOXO3a siRNA transfection (Figure 7G–7I). Together, these data supported our hypothesis that GL-V9 exerted the antioxidative effect by up-regulating Trx-1 via activation of AMPK/FOXO3a pathway.

## DISCUSSION

Ulcerative colitis is a prevalent inflammatory bowel disease in North America and Europe [42]. Nowadays UC is more common even in Asian countries [43]. So far, there are various emerging therapies for UC. Unfortunately, these current treatments for UC, like corticosteroids, sulfasalazine, classical immunosuppressives and antibiotics, are limited in wide clinical applications for their numerous and serious side effects [44, 45]. Hence, novel therapeutic options with high efficacy and safety are urgently required.

The DSS-induced colitis model has the advantage of mimicking human inflammatory bowel disease [46]. We therefore established this model to investigate the protective effect of GL-V9, a newly synthetic flavonoid derivative. As 5-ASA is effective in preventing colitis in humans and animal models [47], we evaluated the efficacy of GL-V9 on colitis using 5-ASA as reference. We selected 50 mg/kg 5-ASA to provide the evidence that 50 mg/kg GL-V9 was effective in DSS-induced colitis. Therefore, GL-V9 doses were 12.5, 25, 50 mg/kg in DSS-induced colitis experiments. In this study, GL-V9 attenuated the acute intestinal injury and inflammatory signs associated with DSS-administration, such as body weight loss, colon length shortening (Figure 1C–1E) and colonic tissue damage (Figure 2A). MPO activity is a marker of neutrophil infiltration, which can be considered as an index of inflammation damage [48]. The enzyme iNOS could regulate NO release which damages the cells of the mucosa and submucosa of the intestine [49]. In our study, GL-V9 decreased MPO and iNOS activities (Figure 2B and 2C). GL-V9 reversed the infiltration of inflammatory cells into colon tissues as well (Figure 2D and 2E). Furthermore, GL-V9 successfully prevented colitis by inhibiting the elevated levels of IL-1β, IL-6 and TNF-α in serum and the high-production of IL-1β, IL-6, TNF-α, MIP-1α and IFN-γ in colons (Figure 3A and 3B). Thus, GL-V9 might be a potential treatment for colitis.

Over the past decades, there has been extensive focus on reactive oxygen species (ROS) as possible etiologic factors in the pathogenesis of intestinal damage in IBD, including UC and CD. The oxidative stress could easily overwhelm the endogenous defenses that regulate ROS production for comparatively low tissue levels of endogenous antioxidants in the colonic mucosa. Therefore, targeting the imbalance between prooxidant and antioxidant mechanisms in IBD may be a promising therapeutic strategy. There are several antioxidative drugs successfully used for ulcerative colitis [50]. Interestingly, our results showed that GL-V9 decreased DSS-induced ROS generation in colons (Figure 3D and 3E). MDA is a very gross indicator of lipid peroxidation induced by ROS, which causes cross-linking of protein and nucleic acid molecules and cell toxicity. Our current data manifested that the elevated MDA content was markedly degraded by GL-V9 (Figure 3G). It has been documented that low endogenous antioxidant defenses, such as glutathione peroxidase (GSH-Px), superoxide dismutases (SOD) are implicated in the intestinal damage in IBD [51]. Glutathione (GSH), the most important intracellular antioxidant defense against oxidative stress, is essential for both the functional and structural integrity of the gut [52]. The sum of all known and unknown endogenous and exogenous antioxidants in a medium is usually called total antioxidant capacity (TAC) and gives a holistic view of antioxidant status. Strikingly, GL-V9 nearly restored the decreased colonic SOD, GSH and total antioxidant capacity to the normal control values (Figure 3F–3I). Our findings indicated that GL-V9 exerted the beneficial effect on DSS-induced colitis via inhibiting oxidative stress and boosting the antioxidant defenses.

AMP-activated protein kinase (AMPK) is well known as an important cellular energy sensor to maintain systemic and cellular energy balance [7]. AMPK regulates cell growth, proliferation and autophagy through modulating protein synthesis by mTOR [53]. mTOR is a central integrator of nutrient that controls cell growth in all eukaryotes which forms two distinct complexes, mTOR complex 1 (mTORC1) and mTOR complex 2 (mTORC2) [54]. mTORC1 recruits downstream substrates such as eukaryotic Initiating Factor 4E Binding Protein 1 (4EBP1) and ribosomal S6 kinase (p70S6K1) that contribute to mTORC1-dependent regulation of protein translation [55]. Activation of AMPK leads to the inhibition of mTORC1. Oncogenic activation of mTORC1 has been reported to promote cell growth and proliferation in multiple types of malignant tumors [56]. However, the physiological role of mTORC1 in experimental mouse models of acute colitis is not fully investigated.

In recent years, emerging evidence indicates that activation of the AMPK pathway reduces intracellular ROS levels to prevent cellular oxidative stress damage [15]. FOXO transcription factors are good candidates regulated by AMPK. The FOXO family of Forkhead transcription factors (FOXO1, FOXO3, FOXO4, and FOXO6 in mammals) plays a crucial role in the regulation of organismal response to oxidative stress, starvation and calorie restriction [57]. FOXO transcription factors integrate cellular signals emanating from insulin, growth factors, cytokines, and oxidative stress [58–60]. The previous study has reported that activation of AMPK induced the nuclear translocation of FOXO3 and the binding of FOXO3 to the Trx promoter [41]. As a major antioxidant protein, thioredoxin-1(Trx-1) could protect cells from ROS-induced cytotoxicity [23]. There is the evidence that human Trx-1 ameliorates experimental murine colitis [30]. Our data demonstrated that GL-V9 remarkably increased phosphorylated-AMPK, Trx-1 expression and nuclear translocation of FOXO3a in the colon tissues of DSS-induced colitis mice (Figure 4A–4G). Moreover, GL-V9 increased the mRNA level of Trx-1 in the colonic tissues of DSS-treated mice (Figure 4H). These results indicated that the protective effect of GL-V9 on DSS-induced colitis was attributed to its antioxidative potential by up-regulating Trx-1 expression via activation of AMPK/FOXO3a pathway.

To confirm the conclusion from our *in vivo* study, we further evaluated the effect of GL-V9 in the mouse macrophage cell line RAW 264.7. In the *in vitro* study, GL-V9 inhibited pro-inflammatory cytokines (IL-1β, IL-6 and TNF-α) and enhanced the antioxidant defenses (Figure 5). As expected, GL-V9 increased phosphorylated-AMPK and Trx-1 expression in RAW 264.7 cells stimulated with LPS (Figure 6A–6C). GL-V9 also remarkably increased nuclear translocation and transcriptional activity of FOXO3a, resulting in up-regulation of Trx-1 transcription (Figure 6D–6J). To further verify the role of AMPK/FOXO3a in anti-oxidative mechanism of GL-V9, we transfected RAW 264.7 cells with AMPK siRNA or FOXO3a siRNA. Trx-1 protein expression increased by GL-V9 was withdrawn by AMPK or FOXO3a siRNA transfection (Figure 7A–7D). AMPK siRNA and FOXO3a siRNA transfection reversed increased transcriptional activity of FOXO3a by GL-V9, respectively (Figure 7F). Furthermore, the inhibitory effects of GL-V9 on LPS-induced ROS generation and pro-inflammatory cytokines production such as IL-1β, IL-6 and TNF-α were remarkably attenuated by AMPK siRNA or FOXO3a siRNA transfection (Figure 7G–7I). Taken together, we hypothesized that GL-V9 exerted the antioxidative effect by up-regulating Trx-1 via activation of AMPK/FOXO3a signaling.

**Figure 8:**
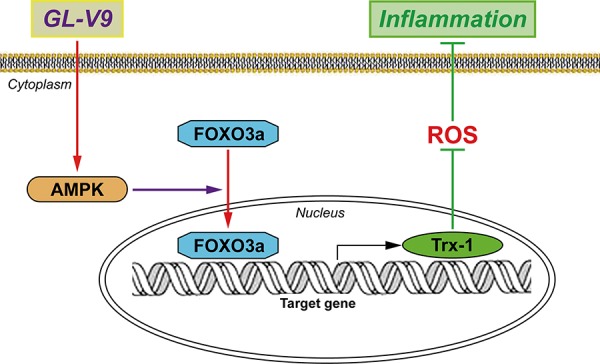
Possible mechanism of GL-V9 inhibits inflammatory responses GL-V9 exerted the anti-inflammation effect by up-regulating Trx-1 via activation of AMPK/FOXO3a pathway.

In conclusion, the present study demonstrated that GL-V9 could alleviate DSS-induced colitis and illustrated its anti-oxidative mechanism by up-regulating Trx-1 via activation of AMPK/FOXO3a pathway (Figure 8). We provided the evidence that maintaining balance of the antioxidant/oxidant system was critical for preventing increased oxidative stress and inflammation in colitis. Our study indicated that GL-V9 might be a potential treatment for ulcerative colitis in humans. However, comprehensive safety assessment and optimized treatment protocol of GL-V9 in clinical applications warrants further research.

## MATERIALS AND METHODS

### Reagents

GL-V9 (C_24_H_27_NO_5_, MW: 409.47), prepared from Dr. Zhiyu Li (China Pharmaceutical University, China), was dissolved in dimethylsulfoxide (DMSO) to 100 mM and stored at −20°C, and freshly diluted with Dulbecco's Modified Eagle Medium (DMEM, GIBCO, Carlsbad, CA) to the final concentration *in vitro* study. *In vivo* study, GL-V9 was prepared as intragastric administration (0.5% sodium carboxyl methyl cellulose (CMC)) by Dr. Xue Ke from college of pharmacy, China pharmaceutical university. The DSS-treated group of mice were administered 0.5% CMC as vehicle.

LPS (E. coli: Serotype O55:B5) and 5-Aminosalicylic acid (5-ASA) were purchased from Sigma-Aldrich (St. Louis, MO). Dextran sulfate sodium (DSS, molecular weight 36–50 kDa) was brought from MP Biomedicals Inc. (Irvine, CA, USA). Diamidino-phenyl-indole (DAPI) were from Sigma (St. Louis, Missouri). Bovine serum albumin (BSA) was purchased from Roche (Mannheim, Germany).

### Antibodies

Antibodies to IL-1β, IL-6, TNF-α and β-actin were from Santa Cruz Biotechnology (Santa Cruz, CA, USA). Antibodies to AMPK, FOXO3a, Lamin A and GAPDH were from Bioworld (Bioworld, Minnesota). Antibodies to Trx-1 was obtained from Abcam (Cambridge, UK). Antibodies to p-AMPK (Thr172) was from Cell Signaling Technology (Danvers, MA, USA). IRDyeTM800 conjugated secondary antibodies were from Rockland Inc. (Philadelphia, PA, USA) and diluted at the ratio of 1:15000.

### Cell culture

The mouse macrophage cell line RAW 264.7 was cultured in Dulbecco's modified Eagle medium (Gibco, CA, USA) supplemented with 10% fetal bovine serum (Gibco, CA, USA), 100 U/ml penicillin and 100 U/ml streptomycin, cells were cultured in a humidified CO_2_ (5%) incubator (Thermo Forma, Waltham, Massachusetts) at 37°C.

### DSS-induced colitis and design of drug treatment

Female C57BL/6 mice, 6–8 weeks old, weighing 18–22 g, were supplied by Shanghai Laboratory Animal Center, China Academy of Sciences. Experimental protocols were in accordance with National Institutes of Health regulations and approved by the Institutional Animal Care and Use Committee. Throughout the acclimatization and study periods, all animals had access to food and water ad libitum and were maintained on a 12 h light/dark cycle (21 ± 2°C with a relative humidity of 45 ± 10%).

Acute colitis was induced by administration of DSS in drinking water. The mice received either drinking regular water (control) or 3% (w/v) DSS drinking water (model) for 7 days and thereafter provided with regular water for 3 days. The mice were randomly assigned to normal, DSS-treated, GL-V9 (12.5, 25 or 50 mg/kg)-treated and 5-ASA (50 mg/kg)-treated groups. GL-V9 and 5-ASA were given intragastrically from day 1 to day 10 respectively.

### Macroscopic assessment and histological analysis of colonic lesions

Animals were weighed daily and inspected for diarrhea and rectal bleeding. For stool consistency, 0 was given for well formed pellets, 2 for loose stools, and 4 for liquid stools. Bleeding was scored 0 for no bleeding, 2 for slight bleeding, and 4 for gross bleeding. After colitis induction animals were sacrificed and colons were removed, opened longitudinally, and washed with phosphate-buffered saline (PBS) and pieces of colonic tissue were used for *ex vivo* analysis. The histological analysis was performed as previously described [34].

### Assessment of myeloperoxidase (MPO) activity

Neutrophil infiltration into inflamed colonic mucosa was quantified by MPO activity assessment using the O-dianisidine method. Proteins extracted from colonic tissues were used to assess MPO levels according to manufacturer's instructions.

### Measurement of iNOS activity

The supernatant of colonic tissue was measured by Nitric Oxide Synthase Assay Kit according to the manufacturer's recommendations.

### Immunofluorescence of colon tissues

CD11b positive inflammatory cell infiltration analysis was performed on paraffin-embedded colon tissue sections. Briefly, the sections were deparaffinized, rehydrated and washed in 1% PBS Tween. Then they were treated with 3% hydrogen peroxide, blocked with 3% bovine serum albumin (BSA) and incubated for 1 h at room temperature with anti-CD11b FITC (1:100). The slides were then counter-stained with DAPI for 30 min. The reaction was stopped by thorough washing in water for 5 min. Images were acquired by confocal laser-scanning microscope (Olympus, Tokyo, JP). Settings for image acquisition were identical for control and experimental tissues.

### Cytokine quantification by enzyme-linked immunoassay (ELISA)

Colons from mice in each group were homogenated with lysis buffer to extract total protein. The homogenate was centrifuged at 12,000 × g at 4°C for 15 min. The amount of total extracted protein was determined by BCA TM protein assay kit (Thermo, MA, USA). The amounts of IL-1β, IL-6, TNF-α, MIP-1α and IFN-γ in the colon homogenate were measured by ELISA kit. IL-1β, IL-6 and TNF-α production in supernatant RAW 264.7 cells and serum of mice were measured by ELISA kits according to the manufacturers' recommendations.

### Immunohistochemistry (IHC)

The expressions of IL-1β, IL-6, TNF-α, p-AMPK and Trx-1 of the colonic tissues was assessed as described in previous study [61].

### Measurement of reactive oxygen species formation

The level of ROS was detected using fluorescent dye 2, 7-dichlorofluorescein-diacetate (DCFHDA, Beyotime Institute of Biotechnology, China). Colons from mice in each group were homogenated with PBS. The colon cells and RAW 264.7 cells were collected and incubated with DCFH-DA for 30 min at 37°C in the dark. The fluorescence intensity was measured using flow cytometry.

### Measurement of colonic glutathione (GSH), superoxide dismutase (SOD) and malondialdehyde (MDA)

Levels of glutathione (GSH) and malondialdehyde (MDA) and the activity of superoxide dismutase (SOD) in colon tissues and RAW 264.7 cells were measured by the kits according to the manufacturer's instructions from Beyotime Institute of Biotechnology (Haimen, China). The total protein content was determined by a bicinchoninic acid (BCA) protein kit (Boster, Wuhan, China) [62].

### Measurement of total antioxidant capacity

Colonic tissues and RAW 264.7 cells were homogenized in cold PBS. The supernatant of colonic tissue was measured according to the kit manufacturer's instructions from Beyotime Institute of Biotechnology (Haimen, China).

### Preparation of cytosolic and nuclear extracts and whole cell lysates

Nuclear and cytosolic protein extracts were prepared according to the modified method as described previously [63]. The cytosolic and nuclear fractions were subjected to immunoblot analysis. The whole cell lysates were prepared as mentioned [64].

### Western blot analysis

After cytosolic, nuclear extracts and whole cell lysates were prepared. Western blot analysis was prepared as described previously [64]. Protein samples were separated by 10% SDS-PAGE and transferred to onto nitrocellulose membranes. The membranes were blocked with 1% BSA at 37°C for 1 h and incubated with indicated antibodies overnight at 4°C, followed by IRDye800 conjugated secondary antibody for 1 h at 37°C. Immunoreactive protein was detected with an Odyssey Scanning System (LI-COR Inc., Lincoln, Nebraska).

### Quantitative real-time PCR analysis

Total RNA isolation and real-time PCR were performed as previously described [61]. The primers used in the reaction were as follows:

Mouse Trx-1-sense (5′-TGCTACGTGGTGTGGAC CTTGC-3′);

Mouse Trx-1- antisense (5′-ACCGGAGAACTCCC CCACCT-3′);

Mouse IL-1β-sense (5′-CCAAGCTTCCTTGTGCA AGTA-3′);

Mouse IL-1β-antisense (5′-AAGCCCAAAGTCCAT CAGTGG-3′);

Mouse IL-6-sense (5′-ACAACCACGGCCTTCC CTAC-3′);

Mouse IL-6-antisense (5′-TCTCATTTCCACG ATTTCCCAG-3′);

Mouse TNF-α-sense (5′-ATGAGCACAGAAAGCA TGATCCGC-3′);

Mouse TNF-α-antisense (5′-AAAGTAGACCT GCCCGGACTC-3′);

Mouse β-actin-sense (5′-TGCTGTCCCTGTATG CCTCT-3′);

Mouse β-actin-antisense (5′-TTTGATGTCACGCA CGCACGATTT-3′).

### Immunofluorescence microscopy

RAW 264.7 cells were pretreated with LPS (1 μg/ml) and GL-V9 (10 μM) for 12 h and then harvested. Cells were fixed with 4% paraformaldehyde in PBS, permeabilized with 0.5% Triton X-100, and blocked with 3% BSA for 1 h. Samples were incubated with primary antibodies (diluted 1:50) against FOXO3a overnight at 4°C. After washed, cells were exposed to FITC-conjugated secondary antibodies (1:1000, Invitrogen, CA, USA, M30101, L42001). Samples were observed and captured with a confocal laser scanning microscope (Olympus Corp., Tokyo, Japan).

### Transient transfection

AMPK siRNA (Santa Cruz, CA) and FOXO3a siRNA (FKHRL1 siRNA) (Santa Cruz, CA) were transfected using Lipofectamine 2000™ reagent (Invitrogen, CA), according to the manufacturer's instructions [65].

### Luciferase assay

A pGMFOXO-Lu (Genomeditech, Shanghai, China), a pRL-TK Renilla (Beyotime, Nan-tong, China) and AMPK siRNA or FOXO3a siRNA were transfected into RAW 264.7 cells using Lipofectamine 2000™ reagent (Invitrogen, CA) [66]. Then cells, lysed by Promega passive lysis buffer, were assayed by using Promega dual luciferase (Firefly luciferase/Renilla luciferase) kit. Luciferase intensity detected with a Luminoskan Ascent (Thermo Fisher Scientific Inc. Finland).

### Statistical analysis

The data shown in the study were obtained in at least three independent experiments and all results represent the mean ± S.E.M. Differences between the groups were assessed by one-way ANOVA and Dunnett's post hoc test. Details of each statistical analysis used are provided in the figure legends. Differences with *P* values < 0.05 were considered statistically significant.

## Abbreviations

5-ASA: 5-Aminosalicylic acid
AMPK: AMP-activated protein kinase
BSA: bovine serum albumin
DAPI: diamidino-phenyl-indole
DCFH/DA: 2′,7′-dichlorofluorescein diacetate
DMSO: dimethylsulfoxide
DSS: dextran sulfate sodium
FOXO: forkhead box subfamily O
GSH: glutathione
IBD: inflammatory bowel disease
IFN-γ: interferon gamma
IL-1β: interleukin-1 beta
IL-6: interleukin-6
iNOS: inducible nitric oxide synthase
LPS: lipopolysaccharides
MDA: malondialdehyde
MIP-1α: macrophage inflammatory protein-1 alpha
MPO: myeloperoxidase
PBS: phosphate-buffered saline
ROS: reactive oxygen species
SOD: superoxide dismutase
Trx-1: Thioredoxin-1
TNF-α: tumor necrosis factor alpha
UC: Ulcerative colitis
